# Amalgam Fillings

**Published:** 1863-06

**Authors:** Geo. Foote


### AMALGAM FILLINGS.

BY GEO. F. FOOTE, M. D.

Not a word have I to offer in favor of amalgams in the mouth. The more I see of them the more I dislike them. They "don't bear acquaintance well;" and though for a season or even for years they may seem to a careless eye not injurious yet in the end they develop mischief. And their mischievousness is of an insiduous character, making its stealthy incroachments upon the general health, demoralizing and disturbing the nervous system and more or less prostrating the whole organism.

Where those fillings are presented to me, my advice in every instance is to remove the amalgams and refill with gold or remove the tooth.

That amalgams do stop external decay in many cases I do not deny. I have witnessed it in a great many instances, but it is the internal mischief that I fear and complain of. Teeth that are the usual recipiants of these fillings are those with large cavities, and the amalgam is brought in close contact with the nervous and circulatory system.

That mercury *per se* is one of the specific poisons particularly obnoxious to the system as is shown in the violent symptoms manifested by the efforts of nature to rid itself of the poison, no one at all conversant with medicine as a science will deny. It is not an element that enters into the formation of any part of the animal body, and its presence is offensive either when taken into the stomach, inhaled through the lungs or left in contact with the surface.

Its disorganization of the osseous system is one of its pathognomonic effects, when taken in too large quantities or repeated too often. That in its crude state or in the form of an amalgam used for filling teeth it would prove highly offensive when suffered to remain in contact with any of the soft parts of the system, no one I think will deny. Now, then, in consideration of these two facts alone, viz: Its specific effect upon the bony structure and its irritating effect upon the soft tissues, it does seem to me that its injurious character, brought in contact as it is with the bony structure of the tooth and from the size of the cavity generally in close proximity and often in contact with its soft parts, is self evident. And when we add to this the constant electrical effect that inevitably follows the conjunction of metals of different affinities, which is constantly being spent upon the nervous system and the objection seems complete against the possibility of an excuse for its use by way of economy or any apparent good.

It is argued by some that the amount of mercury is too small to effect any injury, though the amount, I believe, equals in weight about one-third of the whole.

But suppose it is small, it is the constant presence in contact with sensitive organs producing a constant irritation, that in the end makes it so effective. "A continual dropping in time wears the rock," and this dynamic power in its effect is potentialized in an inverse ratio to the devitalized condition of the parts in contact with and adjacent to the filling, and the longer it remains, the more offensive it becomes on account of the increased susceptibility.

But then the dose is not so very small after all. I have just removed an ordinary one that weighs over 24 grs.; one-third of it is metallic mercury 8 grs.

An ordinary pill of blue mass contains 1 gr. of mercury which is considered an ordinary dose for its alterative effect, four or five produces purgation. The former dose taken daily for some days produces ptyalism and the horrors of a mercurial sore mouth. This, please observe, is the result when taken into the stomach. But what shall be the result when these 8 grs. of mercury are brought directly in contact with the nerves and blood vessels of the jaw walled in as they are by a bony structure?

Just such a result as appeared in this very case and, by the way, this was a modern made filling, the materials of which were prepared and sold at one of our prominent dental depots and inserted by one of our prominent dentists about one year since, viz: Blackening and disintegration of the tooth bone contiguous to the filling beneath the surface, destruction of the nerve, absorption of the fangs, irritation and soreness of the gums, facial neuralgia, great irritability of the nervous system, with the usual concomitant symptoms of all those affections. The whole is better given in the following extract taken from a letter written by a distinguished clergyman living in New York city, who some five years ago consulted me in regard to a tooth with an amalgam filling and at which time I advised its removal. But his reply was that he could not spare it for it had been one of his principle grinders for years. Hear his opinion of it now. "I must tell you about one of the old offenders that twenty-five years ago was filled by Dr. Boynton with an amalgam. I have shown it you several times. Now that it is out I wonder that I ever lived with it in. I am distinctly conscious of the absence of a constant galvanic current that no doubt had plagued me for years and had given me a thousand pains that I had blamed to other causes. The gum and jaw were so diseased where it stood that I came near losing my life by the loss of blood which could not be stayed for about five days. The tooth I have kept to show you. It is charred black as coal. A month has passed and I can hardly realize the change for the better in my feelings."

I have removed a great many amalgams, and I have heard repeated and numerous expressions similar to the above, viz: of the wonderful change in the feelings and improvement in the general health after the removal of amalgam fillings, and many of these same persons had not previously localized the cause of their troubles in the teeth from which the removals had been made.

I am well aware that in offering these opinions I antagonize with many respectable practitioners who honestly differ with me upon this subject, and, with all due regard to their professional experiences, I must utter the truth, as seen from my stand point, and either educate them up to the true light, as I see it, or be myself better posted by an honest discussion of the truth.

